# Inhibition of Extracellular Signal-Regulated Kinases Ameliorates Hypertension-Induced Renal Vascular Remodeling in Rat Models

**DOI:** 10.3390/ijms12128333

**Published:** 2011-11-28

**Authors:** Li Jing, Jianzhong Zhang, Jinping Sun, Fengying Guo, Xin An, Kan Yang, Ping Andy Li

**Affiliations:** 1 Department of Pathology, Basic Medical College, Ningxia Medical University, Yinchuan 750004, Ningxia, China; E-Mails: ljing@nccu.edu (L.J.); sunjp@nxmu.edu.cn (J.S.); guofy@nxmu.edu.cn (F.G.); 2 Department of Pharmaceutical Sciences, Biomanufacturing Research Institute and Technology Enterprise (BRITE), North Carolina Central University, Durham, NC 27707, USA; 3 Department of Pathology, First Hospital of Handan, Handan 056002, Hebei, China; E-Mail: anxin331@126.com; 4 Department of Ophthalmology, Lanzhou First People’s Hospital, Lanzhou 730000, Gansu, China; E-Mail: gslzyk@126.com

**Keywords:** artery, extracellular signal-regulated kinase, hypertension, kidney SHR, vascular remodeling

## Abstract

The aim of this study is to investigate the effect of the extracellular signal-regulated kinases 1/2 (ERK1/2) inhibitor, PD98059, on high blood pressure and related vascular changes. Blood pressure was recorded, thicknesses of renal small artery walls were measured and ERK1/2 immunoreactivity and *erk2* mRNA in renal vascular smooth muscle cells (VSMCs) and endothelial cells were detected by immunohistochemistry and *in situ* hybridization in normotensive wistar kyoto (WKY) rats, spontaneously hypertensive rats (SHR) and PD98059-treated SHR. Compared with normo-tensive WKY rats, SHR developed hypertension at 8 weeks of age, thickened renal small artery wall and asymmetric arrangement of VSMCs at 16 and 24 weeks of age. Phospho-ERK1/2 immunoreactivity and *erk2* mRNA expression levels were increased in VSMCs and endothelial cells of the renal small arteries in the SHR. Treating SHR with PD98059 reduced the spontaneous hypertension-induced vascular wall thickening. This effect was associated with suppressions of *erk2* mRNA expression and ERK1/2 phosphorylation in VSMCs and endothelial cells of the renal small arteries. It is concluded that **i**nhibition of ERK1/2 ameliorates hypertension induced vascular remodeling in renal small arteries.

## 1. Introduction

Elevated arterial blood pressure is known to induce vascular structural change, which is termed as vascular remodeling (VR). VR mainly involves thickening and stenosis of the vascular wall. Previous studies have suggested that VR is not only a pathophysiological basis for the progression of hypertension but also for the development of other cardiovascular diseases [1]. Multiple factors, including blood pressure, oxidative stress, extracellular matrix and vascular smooth muscle cells (VSMCs) influence VR [2]. Modulating these factors could reduce or even reverse VR, thereby suppressing the progression of hypertension. For example, angiotensin-converting enzyme inhibitor (ACEI) has been shown to reduce blood pressure and to reverse the cardiovascular remodeling [3]. Similarly, anti-oxidants ameliorate VR through reducing production of free radicals and inhibiting oxidation of low-density lipoproteins [4]. Reversal of VR using pharmacological or genetic approaches may hold great potential for treating hypertension and hypertension-induced cardiovascular disorders.

VSMCs remodeling in vascular wall during hypertension is mediated by activation of cell signal transduction pathways, such as protein kinase C (PKC) and mitogen-activated protein kinase (MAPK). Activation of these pathways results in proliferation of VSMC, eventually causing an increase in cell numbers or alteration of VSMC function [5]. Extracellular signal-regulated kinases 1 and 2 (ERK1/2) of MAPK family are core factors that regulate cell hypertrophy and proliferation [6,7]. Although, it is already known that ERK1/2 MAPK signal transduction cascade plays a key role in modulating VSMC proliferation, it remains unknown whether inhibition of ERK1/2 MAPK signaling pathway can ameliorate the VR process or even reduce blood pressure in hypertensive animals. The objective of this study was to explore the effects of ERK1/2 inhibitor PD98059 on VR and blood pressure in spontaneous hypertensive rats (SHR). The results revealed that while inhibition of ERK1/2 ameliorated the progression of VR, it failed to reduce blood pressure of SHR.

## 2. Results and Discussion

### 2.1. Physiological Variable

**Figure 1:**
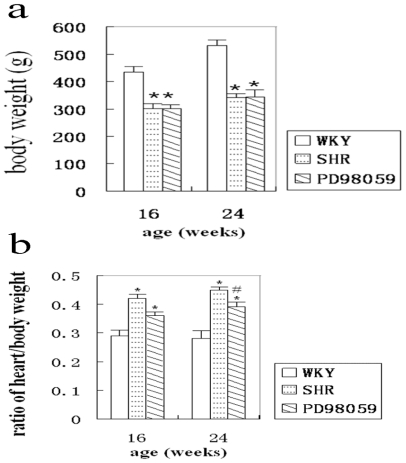
Body weight (**a**) and heart and body weight ratio (**b**) in normotensive WKY rats, SHR and PD98059-treated SHR at 16 and 24 weeks of age.* *P* < 0.05 *vs.* WKY control and ^#^ *P* < 0.05 *vs.* SHR group.

All animals survived to the pre-determined end-point. Body weight of non-treated hypertensive rats was significantly reduced compared with WKY rats (435.11 ± 41.86 g *vs.* 531.63 ± 43.38 g). The body weight of PD98059-treated hypertensive rats was 302.60 ± 13.87 g at 16-weeks and 344.17 ± 22.23 g at 24-weeks, which was significantly lower than that of the age-matched WKY group (Figure 1a). Cardiac mass was determined in each animal and heart over body weight ratio was calculated. This ratio in SHR was 0.42 ± 0.20 at 16-weeks and 0.45 ± 0.20 at 24-weeks of age, which was significantly higher than that of the age matched WKY group (0.29 ± 0.10 and 0.28 ± 0.20 for 16- and 24-weeks of age, respectively). Treatment with PD98059 moderately reduced the heart/body ratio at 16- and 24-weeks, although only the values at 24-weeks reached statistical significance (Figure 1b).

**Figure 2:**
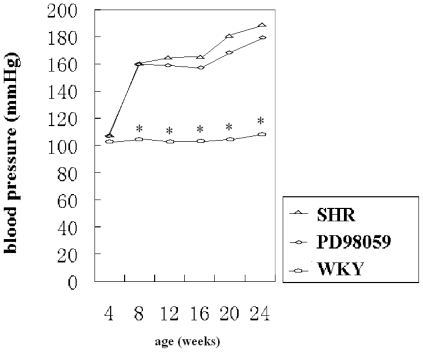
Arterial blood pressure in control SHR, PD98059-treated and normotensive WKY rats. Blood pressure increased in SHR and PD98059 groups. * *P* < 0.05 *vs.* SHR and PD98059 groups.

Arterial blood pressure remained normal in normotensive WKY rats at 16- and 24-weeks (102.50 ± 11.26 mmHg to 108.70 ± 8.48 mmHg, respectively). In SHR, blood pressure was normal at 4 weeks (108.10 ± 7.09 mmHg), elevated at 8 weeks and remained hypertensive at 16- and 24-weeks compared with age-matched WKY controls (Figure 2) Treatment with PD98059 failed to reduce blood pressure, therefore, no difference in blood pressure was detected between the PD98059 group and age-matched SHR group.

### 2.2. Vascular Wall Morphology

**Figure 3:**
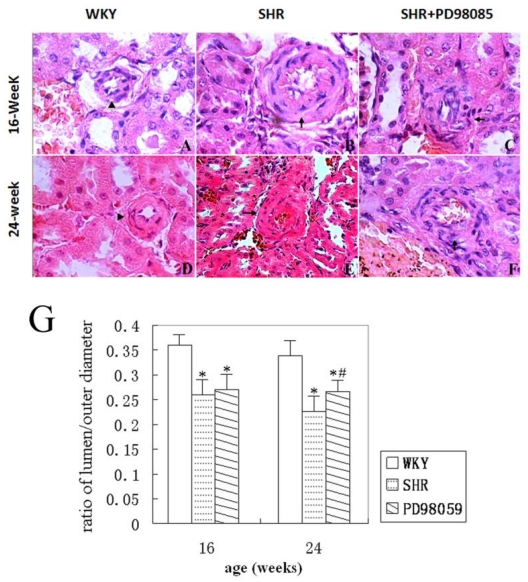
Morphological changes of renal interlobular arteries. (A–F) representative histograms of renal interlobular arteries. (**A**) normal morphology of WKY rats at 16 weeks of age group (▴); (**B**) increased thickness of interlobular artery wall in SHR at 16 weeks of age group (↑); (**C**) asymmetrically arranged VSMCs in PD98059-treated SHR at 16 weeks of age group (↑); (**D**) normal morphology of WKY rats at 24 weeks of age group (▴); (**E**) increased thickness of vessel wall in SHR at 24 weeks of age group (↑); (**F**) irregular arranged VSMCs and thickened wall in PD98059-treated SHR at 24 weeks of age (↑). H&E staining. Magnification, 400×. (**G**) Summarized ratio of the inner to outer diameter of the renal small arteries from 3 groups of animals. * *P* < 0.05 *vs.* WKY group and ^#^ *P* < 0.05 *vs.* SHR group.

The structure of renal arteries and arterioles were examined on histological sections. The inner and outer diameters of renal arteries of transverse sections were measured and the thickness of the vascular wall was calculated by subtracting the inner diameter from the outer diameter. No abnormal arterial wall change was observed at WKY rats (Figure 3A,D). Renal arteriole structures were normal at 4 weeks old SHR (data not shown). However, arteriole wall thickening, as reflected by a decreasing ratio of vascular inner to outer diameters, and luminal stenosis were observed in small arteries, especially the interlobar arteries, at 16 weeks old SHR (Figure 3B,G). At 24 weeks, additional changes, including proliferation and asymmetric arrangement of VSMC, as well as separation and breakage of internal elastic membrane were detected in arch arteries and interlobular arteries of the SHR (Figure 3E). Treatment with PD98059 failed to prevent the vascular structural alterations observed in SHR at 16 weeks (Figure 3C). However, PD98059 significantly ameliorated the thickening of the vascular wall at 24 weeks (Figure 3F), as reflected by the increased ratio of the inner to outer diameter of the vascular wall. Representative histograms of interlobar arteries are given in Figure 3A–F and the summarized ratio of renal interlobar arteries from 3 groups of rats is presented in Figure 3G.

### 2.3. ERK Protein and mRNA Expression

**Figure 4:**
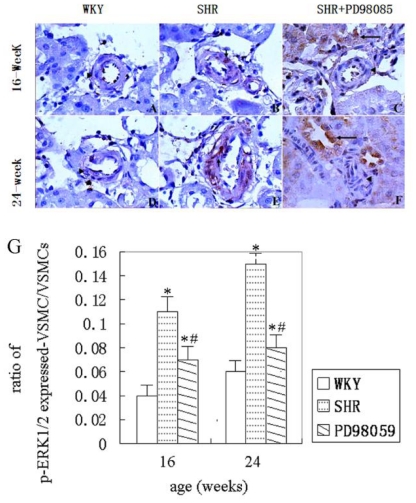
Phospho-ERK1/2 immunohistochemistry in VSMCs of interlobar arterioles. **A**–**F**, representative ERK1/2 immunohistostaining in VSMCs. (**A**) no expression of phospho-ERK1/2 in WKY at 16 weeks of age group (▴); (**B**) increased expression of phospho-ERK1/2 in SHR at 16 weeks of age group (↑); (**C)** reduced expression of phospho-ERK1/2 in PD98059-treated SHR at 16 weeks of age group (↑); (**D)** no expression of phospho-ERK1/2 in WKY rats at 24 weeks of age group (▴); (**E)** increased expression of phospho-ERK1/2 in SHR at 24 weeks of age group; (**F**) reduced expression of phospho-ERK1/2 VSMC except distal renal tubule (↑) in PD98059-treated SHR at 24 weeks of age group (▴). DAB visualized, slight hematoxylin counterstaining. Magnification, 400×. **G**, Summary of phospho-ERK1/2 positively stained VSMCs in the small renal arteries. * *P* < 0.05, *vs.* WKY group; ^#^ *P* < 0.05, *vs.* SHR group.

Phosphorylated ERK-1/2 in VSMCs of the renal small arterioles was detected by immunohistochemical staining. Representative histograms are given in Figure 4A–F and summarized data are presented in Figure 4G. As shown in these figures, there was no phospho-ERK1/2 immunoreactivity detected in normotensive WKY rats at either 16 or 24 weeks. A few positively stained renal VSMCs were observed in SHR at 16 weeks and the number increased at 24 weeks. As shown, the ratio of phospho-ERK1/2 positive-stained VSMCs to total VSMCs significantly increased at both 16 and 24 weeks of age compared with normotensive WKY rats. Treatment of PD98059 in SHR group significantly reduced the number of phospho-ERK1/2 positive renal small artery VSMCs at both 16 and 24 weeks of age *(P* < 0.05).

**Figure 5:**
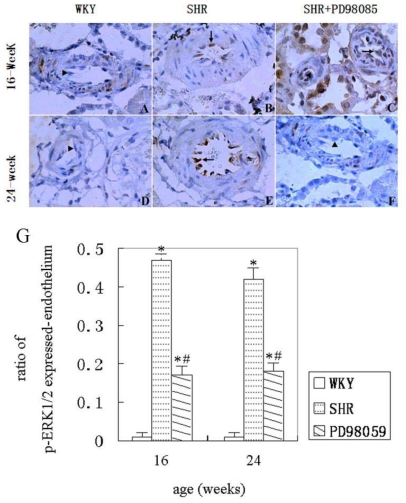
Phospho-ERK1/2 immunohistochemistry in endothelial cells of the small renal arteries (A–F). (**A**) no expression of phospho-ERK1/2 in WKY rats at 16 weeks of age group (▴); (**B**) increase in number of phospho-ERK1/2 positive cells in SHR at 16 weeks of age group (↑); (**C**) reduction in number of phospho-ERK1/2 positive cells in PD98059-treated SHR at 16 weeks of age group (↑); (**D**) no expression of phospho-ERK1/2 in WKY rats at 24 weeks of age group (▴); (**E**) increase in number of phospho-ERK1/2 positive cells in SHR at 24 weeks of age group; (**F**) significantly decrease in number of phospho-ERK1/2 positive cells in PD98059-treated SHR at 24 weeks of aged group (↑). DAB was used as substrate. Hematoxylin counterstaining. Magnification, 400×. (**G**) summarized data showing number of phospho-ERK1/2 positive endothelial cells of the small renal arteries. * *P* < 0.05 *vs.* WKY group and ^#^ *P* < 0.05 *vs.* SHR group.

Similar changes were also observed in endothelial cells of the renal small arteries. Therefore, few or no immunoreactivity of phospho-ERK1/2 was detected in normotensive WKY rats at 16 and 24 weeks. The number of phospho-ERK1/2 positive endothelial cells increased significantly at 16 and 24 weeks in SHR animals. Treatment with PD98059 in SHR significantly reduced the number of phospho-ERK1/2 positive cells at 16 and 24 weeks. A set of representative histograms showing phospho-ERK1/2 immunohistology in endothelial cells is given in Figure 5A–F and summarized data are presented in Figure 5G.

**Figure 6:**
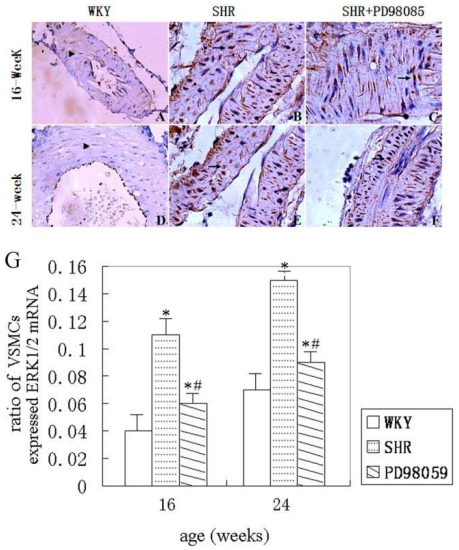
A set of histograms showing *in situ* hybridization of *erk2* in VSMCs of small renal arteries. Little to none *erk2* mRNA expression (▴) was observed in WKY rats at 16 and 24 weeks (**A**,**D**). Marked increase of *erk2* mRNA expression (↑) was detected in SHR at 16 and 24 weeks of age group (**B**,**E**). *Erk2* mRNA was reduced in PD98059-treaetd SHR (↑) at 16 and 24 weeks of age group(**C**,**F**). Arrow heads denote negative cells and arrows denote positive cells. DAB was used as substrate. Sections were counterstained with hematoxylin. Magnification, 400×. (**G**) summarized bar graph showing number of *in situ* phospho-ERK1/2 VSMCs over total observed VSMCs in three groups of animals at 16 and 24 weeks. * *P* < 0.05 *vs.* WKY group and ^#^ *P* < 0.05 *vs.* SHR group.

*Erk2* mRNA expression was detected using *in situ* hybridization. The results showed that little or non-detectable mRNA expression in WKY rats at 16 and 24 weeks of age (Figure 6A,D). *Erk2* mRNA expression was markedly enhanced in VSMCs of the small renal arteries of SHR at 16 and 24 weeks (Figure 6B,E), as well as in epithelial cells of renal distal convoluted tubule (data not shown). Treating SHR with PD98059 significantly suppressed the *erk2* mRNA expression in the VSMCs of the small renal arteries (Figure 6C,F). A set of histograms showing *erk2 in situ* hybridization in small renal arteries are given in Figure 6A–F and the summarized data are presented in Figure 6G.

### 2.4. Discussion

Chronic hypertension changes the dimensions and properties of arterial wall. These changes include proliferation of VSMCs and thickening of arterial wall (for review see [8]). One of the molecular mechanisms responsible for vascular remodeling is activation of ERK1/2, which causes VSMCs to proliferated and deposit collagen type I on microvessels [9,10]. Our results showed that spontaneous hypertension increased the thickness of renal small artery wall and the proliferation of VSMCs. In agreement with previous studies [7,11], phosphor-REK1/2 MAPK immunoreactivity enhanced in renal VSMCs and endothelial cells. Inhibition of ERK1/2 using PD98059 suppressed ERK1/2 immunoreactivity and ameliorated the thickening of vascular wall in 24 weeks SHR. Our study demonstrated that inhibition of ERK1/2 kinases could significantly suppress the progression of vascular remodeling in spontaneous hypertensive animals.

In the present study, SHR developed hypertension at 8 weeks of age, which is in agreement with previous published report [7,12] Hypertension increases peripheral blood vessels resistance through vascular remodeling, a process involving VSMC proliferation, hypertrophy, blood vessel compliance reduction and the narrow vessel lumen. The decreased body weight in SHR compared with WKY rats in the present study reflects the general deterioration of health associated with hypertension. The increased heart to body mass ratio in SHR reflects myocardial hypertrophy in response to increase cardiac after-load caused by high blood pressure.

VSMCs regulate vascular structure and reactivity under physiological and pathological conditions [13–15]. Normally, VSMCs are in a non-proliferative state. After being stimulated by stretch or high blood pressure, VSMCs proliferate, vascular smooth muscle layer become thicker and vascular lumen size decreases. As results, vascular resistance increases [16]. Several factors regulate the VSMCs, including platelet-derived growth factor, insulin-like growth factor, norepinephrine, endothelin angiotensin II and cytokines [3,5–7,17]. These factors are released during vascular injury. They convert VSMCs from quiescent state to active synthetic state [18,19]. Activated VSMCs synthesize and release vasoactive factors, growth factors and extracellular matrix proteins, which cause VSMC hypertrophy, proliferation and migration, excessive accumulation of extracellular matrix, and eventually, vascular remodeling [20,21]. Abnormal proliferation of VSMC is a common pathophysiological change observed in hypertension [21]. The increase in number of VSMCs and narrowingness of renal arterioles observed in the present study reflect vascular remodeling induced by hypertension. Such remodeling is likely mediated by ERK1/2.

Activation of ERK1/2 by phosphorylation causes cell proliferation and growth [22–24]. ERK1/2 is activated by MAPK kinase (MEK) and the latter is activated by Raf-1. The ERK1/2 MAPK signaling pathway plays an important role in cell proliferation and extracellular matrix deposition during hypertensive cardiovascular remodeling [25]. ERK1/2 activation stimulates vascular smooth muscle cell hypertrophy and hyperplasia, which lead to an increase in peripheral resistance and elevation of blood pressure. In present study, ERK1/2 phosphorylation increased significantly in the endothelial cells and VSMCs of the small renal arterioles in SHR at 16 and 24 weeks, suggesting that ERK1/2 was involved in the hypertension-induced vascular remodeling. The involvement of ERK1/2 in hypertension-induced renal vascular remodeling was further supported by the results obtained from ERK1/2 inhibitor studies.

Treating SHR with ERK1/2 inhibitor PD98059 significantly reduced the number of VSMCs and increased the lumen size of small renal arterioles after 4–8 weeks. These effects are associated with suppression of phospho-ERK1/2 levels in the endothelial cells and VSMCs. Since inhibition of ERK1/2 resulted in suppression of vascular remodeling in SHR, the results may suggest that hypertension-induced vascular remodeling is mediated through ERK1/2 signaling pathways. Furthermore, our data also suggest that inhibition of ERK1/2 ameliorates hypertension-induced vascular remodeling. Inhibition of ERK1/2 has been shown to reduce blood pressure in leptin-induced hypertension [26] and in endothelin-induced hypertension [27]. Our results showed that inhibition of ERK1/2 failed to lower blood pressure in SHR animals, indicating PD98059 may not be used as a blood pressure lowering drug. However, since PD98059 decelerated the development of the renal vascular remodeling, it may be effective in reducing the progression of hypertension and in preventing further blood pressure increase in SHR animals. Combination of blood lowering drug with ERK inhibitor may be beneficial to hypertensive patients.

## 3. Materials and Methods

### 3.1. Animals and Reagents

Sixty-four SHR rats (16 males and 16 females) and 8 normal WKY (4 males and 4 females) were purchased from Vitalriver Experimental Animal Technology Co., Ltd. (Beijing, China). All animal care and procedures were in strict accordance with the China Laboratory Animal Use Regulations and were approved by the Institutional Animal Care and Use Committee at Ningxia Medical University, Yinchuan, China. Efforts were made to minimize animal numbers and stress. Monoclonal anti-phospho-ERK1/2 antibody (Cell Signaling), ERK1/2 inhibitor PD98059 (Merck), Streptavidinbiotin complex kits (Zymed), and protein markers were purchased from Wuhan Boster Biological Engineering Limited (Wuhan, China).

### 3.2. Animal Treatments

Animals at 4 weeks of age were randomly divided into 3 groups: (i) Normotensive WKY rats (WKY group, *n* = 8), (ii) SHR control group (SHR group, *n* = 16), and (iii) PD98059-treated SHR group (PD98059 group, *n* = 16). Animals in the treatment group were intraperitoneally injected with PD98059 (10 mg/kg) once a day for 5 days per week, while those in SHR group and WKY group were injected with 0.9% saline. The injections were lasted for 12 or 20 weeks. Blood pressure was monitored once a week using a tail BP-6 animal non-invasive blood pressure device (Chengdu China) during the experimental period. Animals were anesthetized using intraperitoneally injection of 4% sodium pentobarbital (30 mg/kg) at the ages of 16 or 24 weeks. The right kidney was harvested. The kidneys were placed in 4% paraformaldehyde for hematoxylin and eosin staining, *in situ* hybridization and immunohistochemistry.

### 3.3. Immunohistochemistry

Immunohistochemistry was performed on kidney sections (5 μm thickness). The sections were treated with 3% H_2_O_2_ for 10 minutes at room temperature to quench endogenous peroxidase activity. Antigen retrieval was performed by placing the sections in citrate buffer and heated for 30-s in a microwave oven before nonspecific binding sites were blocked in 5% bovine serum albumin (BSA) in PBS/0.2% TX-100 for 30 min. The sections were incubated in primary anti-phospho-ERK1/2 antibody (1:400 dilution) overnight at 4 °C. After 3 washes in phosphate buffered saline (PBS), the sections were then incubated with biotinylated secondary antibody for 30 min at room temperature. The a vidin-biotin complex (ABC) technique was then applied. Sections were stained in a horseraddish peroxidase substrate solution (diaminobenzidine), counter-stained with hematoxylin and mounted on glass slides. The sections were scanned using a computer-assisted imaging system (Zeiss LSM5 Image Examiner software). The inner and outer diameters of small renal arteries were measured using image analysis software (Zeiss LSM5 Image Examiner software). Numbers of ERK1/2 positive stained VSMCs and endothelial cells were counted on a 50 mm^2^ on each section using a grid ruler placed in the optical lens and the result is presented as a ratio of positive cells to total cells in the area.

### 3.4. In Situ Hybridization

Digoxygenin-labeled riboprobes that were synthesized by Wuhan Boster Biological Engineering Limited (Wuhan China) were used for the detection of rat ERK2 mRNA. The oligonucleotide probe sequences are as follows: Sense: 5′-AGATG GTCCG CGGGC AGGTG TTCGA CGTAG-3′ and antisense: 5′-AAAAT AAGGT GCCAT GGAAC AGGTT GTTCC-3′. The probe specificity was confirmed by BLAST software testing.

After deparaffinizaton, rehydration and quench of endogenous peroxidase activity by 3.0% H_2_O_2_ at room temperature for 5–10 min, the sections were treated with pepsin freshly dissolved in 3% citric acid solution for 25 min at 37 °C incubator. Followed fixed with 1% paraformaldehyde for 10 min at room temperature and incubated with 20 μL of pre-hybridization solution for 3 hours at 38–42 °C, the sections were incubated with sense or anti-sense (control) riboprobe hybridization solution overnight at 38–42 °C. The reaction was stopped by dropping blocking solution on the slides. Biotinylated mouse anti-digoxigenin was applied on the sections for 60 min at 37 °C, and followed by reaction of SABC. Biotin-peroxidase complex was applied and diaminobezidine was used as a substrate. The sections were counterstained with hematoxylin and mounted. The images were analyzed under a microscope and presented as a ratio of positively stained cells to total cells for a 50 mm^2^ area.

### 3.5. Statistical Analysis

The values were expressed as means ± SEM. ANOVA followed by Tukey’s test was used to analyze the differences among 3 groups. Statistical significance was determined as *P* < 0.05.

## 4. Conclusion

Our study showed that spontaneous hypertension increased the thickness of the renal arterial wall at 16 and 24 weeks compared with normotensive WKY rats. In addition, hypertension increased both phosphorylation of ERK1/2 and mRNA expression of *erk2* in renal small arterial endothelial cells and VSMCs at 16 and 24 weeks of age. Furthermore, our results demonstrated that inhibition of ERK1/2 activity by PD98059 in hypertensive rats significantly reduced the *erk2* mRNA expression, inhibited ERK1/2 phosphorylation and ameliorated the hypertension-induced vascular alterations. These results suggest that hypertension-induced renal vascular remodeling is likely mediated by the ERK1/2 signaling pathway.
